# Homolytic fracture of inorganic crystalline materials enhances the mechano-chemical degradation of polypropylene

**DOI:** 10.1039/d5sc03348a

**Published:** 2025-08-15

**Authors:** Adrian H. Hergesell, Stephan Popp, Raghavendra Meena, Viviana M. Ospina Guarin, Claire L. Seitzinger, Carsten Sievers, Guanna Li, Ina Vollmer

**Affiliations:** a Inorganic Chemistry and Catalysis Group, Institute for Sustainable and Circular Chemistry, Utrecht University The Netherlands i.vollmer@uu.nl; b Biobased Chemistry and Technology, Wageningen University The Netherlands; c School of Chemical & Biomolecular Engineering, Georgia Institute of Technology Atlanta Georgia USA

## Abstract

Mechano-chemistry can depolymerize plastics to their monomers. The conversion of polyolefins, however, suffers from low chain cleavage rates and the low stability of radical intermediates. Therefore, insights into the degradation mechanism are crucial to obtain higher yields. Herein, we promote the mechano-chemical degradation of polypropylene by milling with sand as an additive, which increases depolymerization yields by a factor of 25. Fracture of sand crystals causes homolytic cleavage of Si–O bonds resulting in unpaired surface electrons, which possess radical reactivity and can initiate degradation reactions of polypropylene, ultimately resulting in smaller hydrocarbons. We show that this mechanism based on surface radicals dominates over alternative pathways based on locally increased temperature or surface roughening of grinding spheres. While inorganic materials, such as glass fiber in composites, are typically unwanted in (chemical) recycling scenarios, we show that they can be exploited to drive mechano-chemical depolymerization. Our study illustrates that control over the radical-based degradation mechanism during the mechano-chemical conversion of polyolefins is key to increase yields and technological viability.

## Abstract 2

The fracture of sand and quartz during ball milling generates surface radicals. These highly reactive species can be used to initiate the mechano-chemical radical depolymerization of polypropylene.

## Graphical abstract

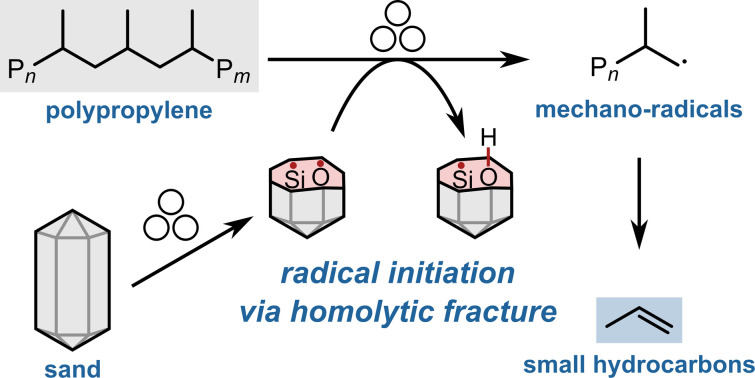

## Introduction

Chemical recycling techniques convert plastic materials into chemicals which can be used as feedstock in the chemical industry, for example for making high-quality plastic items again.^1,2^ Polyethylene and polypropylene (PP), which are the most produced plastics worldwide, can be pyrolyzed.^1^ During this treatment under an inert atmosphere, high temperatures induce random cracking into smaller gaseous, liquid, and solid hydrocarbons. However, due to the combination of a high temperature and the high energy of cracking intermediates, control over the product selectivity is difficult to achieve. Therefore, typical pyrolysis oils contain a multitude of different hydrocarbon species and need further energy intensive upgrading.^3^ Alternative low-temperature strategies could help to gain control over side reactions and to lower the energy demand.

Mechano-chemical bond scission of polymers induced, *e.g.*, during ball milling, has been reported at temperatures as low as −196 °C,^4,5^ and has recently been exploited for the purposeful depolymerization of polystyrene derivatives,^6–8^ polyacrylates,^9^ and polyolefins.^10,11^ The activation step is the homolytic backbone scission occurring under impact of the grinding spheres in the ball mill. The cleavage generates a pair of mechano-radicals, which can depolymerize according to established radical-based mechanisms, leading to monomers *via* β scission or more complex (side) products after radical transfer reactions.^12^ The carbon-centered radicals usually have a short lifetime and react with one another to terminate.^12^ Controlling the radical pathway during mechano-chemical conversion recently enabled us to increase small hydrocarbon yields significantly by stabilizing intermediates *via* mechano-catalysis.^10^ Besides thermodynamic limitations,^13^ however, total yields were limited also by the slow reaction initiation *via* chain cleavage compared to fast deactivation of resulting radicals.^10,14^ Further insights into the interplay of initiation, stabilization, (de)propagation and termination steps will help to overcome this barrier. To this end, materials that can provide additional radical functionality, and can thereby initiate reactivity during mechanical activation, are promising additives.

The fracture of certain materials in which atoms are connected by covalent rather than ionic or metallic bonds can cause the presence of surface radicals by homolytic σ bond cleavage.^15^ Such substances include SiO_2_, nitrides (BN, Si_3_N_4_) or covalent elements (Si, C).^15^ In the case of SiO_2_, for example, fracture forms silyl (≡Si˙) and siloxyl (≡Si–O˙) radical sites on the newly generated surfaces.^15^ These are formed *via* homolytic cleavage of Si–O bonds, which modelling showed to be favored compared to heterolytic cleavage.^16^ Due to their high concentration and energies, such radical surface species have been described as a surface plasma, although their nature should not be confused with true plasma states.^15,17^ The unpaired nature of their electrons allows the observation of such radical species *via* electron paramagnetic resonance (EPR) spectroscopy. More fracturing events lead to higher amounts of radicals detected.^18,19^ In addition, these surface sites possess radical reactivity that can be exploited for chemical transformations.^15^ Fractured SiO_2_ reacts with O_2_ and N_2_ to form nitrogen oxides.^15^ SiO_2_ also reacts with organic compounds, for example in the radical-based degradation of methane and organic environmental toxins.^15,17^ Furthermore, ball milling of SiO_2_ generates surface radicals which readily abstract hydrogen atoms from ethanol, and the free radical 2,2-diphenyl-1-picrylhydrazyl is quenched when milled together with SiO_2_ and ethanol.^20–22^ Other examples are the polymerization of ethylene and acrylic acid derivatives enabled by the fracture of alumina,^23,24^ while grinding SiO_2_ can initiate the polymerization of methyl methacrylate.^25^ Nowadays, in an attempt to develop chemical conversion methods for plastics, the reverse reaction of such polymerizations is desired.^1^ Therefore, we herein investigate the reactivity of surface radicals, generated during milling of SiO_2_-based materials, towards the depolymerization of PP.

## Results and discussion

### Milling with sand boosts mechano-chemical depolymerization

We investigated the influence of crystalline powder additives on the mechano-chemical conversion of PP into small hydrocarbons using a shaker mill. Five 10 mm ZrO_2_ grinding spheres were used at 30 Hz to mill typically 2 g of PP and 1 g of additive. The sand used herein is fine quartz sand with particle sizes of <500 μm composed of crystalline α-quartz (see Fig. S1A for scanning electron microscopy (SEM) and Fig. S2 for X-ray diffractograms). A gas in- and outlet was added to a commercial 25 ml ball mill vessel to allow for the continuous analysis of gaseous products using a gas chromatograph. The products of interest are gaseous hydrocarbons, especially the monomer propene, which are sufficiently volatile and allow for time-resolved analysis.^13^ Heavier hydrocarbons are not listed as reaction products, as they originate, at least partially, from additives which interferes with a reliable measurement of their mechano-chemical formation rates.^26^

**Fig. 1:**
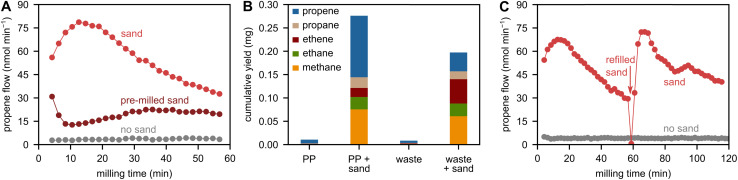
(A) Propene flow during milling of 2 g of PP with 5 ZrO_2_ spheres at 30 Hz with and without 1 g of sand (pristine and pre-milled). (B) C_1–3_ hydrocarbon yield obtained after 1 h of milling 2 g of PP or 1 g of industrial foil waste with 5 ZrO_2_ spheres at 30 Hz with and without 1 g of sand. (C) Propene flow during milling of 2 g of PP with 5 ZrO_2_ spheres at 30 Hz with and without sand. 1 g of sand was used initially, and 1 g of sand was re-filled at the indicated point in time.

Milling PP with ZrO_2_ grinding spheres produces small amounts of hydrocarbons, such as the monomer propene (Fig. 1A) *via* a radical pathway consisting of backbone cleavage and subsequent depolymerization.^10,12,27^ When milling with sand, the cumulative yield is boosted by a factor of 24.9 ± 1.2 (Fig. 1B, see Fig. S3 for repetitions). Decreasing the PP loading to 0.1 g yields 1.33 wt% C_1–3_ hydrocarbons when milling for 30 min with sand, while only 0.14 wt% are obtained without (Fig. S4). Beyond C_3_ hydrocarbons, we observed products up to C_10_ when ball milling PP without sand, and the formation of the C_5_ fraction is boosted when adding sand (Fig. S4). The addition of sand also boosts hydrocarbon productivity when milling industrial foil waste which represents a realistic waste stream (Fig. 1B). Generally, adding sand shifts the selectivity to more saturated products. This could be rooted in an increase in radical transfer reactions leading to saturated hydrocarbons as opposed to direct depolymerization of the radical chain ends *via* β scission leading to propene. The production rate curves of small hydrocarbons when milling PP with sand all have similar features: There is an induction period leading to peak productivity after *ca.* 10 min, and activity declines steadily afterwards. Milling longer reveals a total loss of activity after *ca.* 240 min (Fig. S5).

Ball milling is a dynamic process with breakage and sintering of particles. During milling, sand is broken down from particle sizes of >200 μm to <10 μm (see Fig. S1B), and we believe that the decrease in sand particle size is connected to the decline in small hydrocarbon formation. This is in line with observations during the decomposition of organic compounds over surface radical-generating materials, since reactivity declines when the particle size of the additive is too small for particles to effectively crack.^15^ To address this limitation in a future application, mixtures of larger and smaller grinding spheres could be used to incur more sand particle breakage down to a high fineness, which would extend reactivity, while also achieving high total energy inputs. Alternatively, mixtures of different milling body geometries could be used.^28^

To experimentally investigate deactivation by particle shrinkage, we pre-milled sand to decrease its particle size from >200 μm to <10 μm (Fig. S1C), and used the resulting material in depolymerization experiments with PP. Using pre-milled instead of pristine sand causes much lower flows of propene and other hydrocarbons (Fig. 1A), showing that sand particles above a critical size are necessary to obtain sufficient depolymerization rates. In addition, using Fe spheres compared to ZrO_2_ spheres causes activity during milling of pristine sand to decline more steeply (Fig. S6). This is likely caused by faster breakage of sand due to the more energetic impacts when using the denser Fe spheres rather than less dense ZrO_2_. This finding illustrates that the balancing of material fracture resistance and impact energies is important for controlled and sustained radical generation.

After activity has declined due to loss in particle size, fresh sand can be refilled to restore productivity (Fig. 1C). However, this approach is not feasible indefinitely due to the accumulation of sand and the depletion of plastic in the milled mixture. Furthermore, the additional damping, often termed cushioning effect, caused by the extra material prohibits sufficiently forceful impacts on the plastic and/or sand particles. Large sand particles are effectively a sacrificial reagent, and the regeneration of large particles has to be considered for a future application. For some applications, such as the decomposition of environmental toxins over fractured quartz, melting together of the resulting powder was suggested to obtain particles large enough to be cleaved again.^15,29^

In addition to sand, the milling of covalent SiC and B_4_C was found to promote the production of small hydrocarbons from PP. Interestingly, production rates are initially lower but persist for longer compared to milling with sand which is likely caused by the higher fracture resistance of SiC and B_4_C (Fig. S7).

**Fig. 2:**
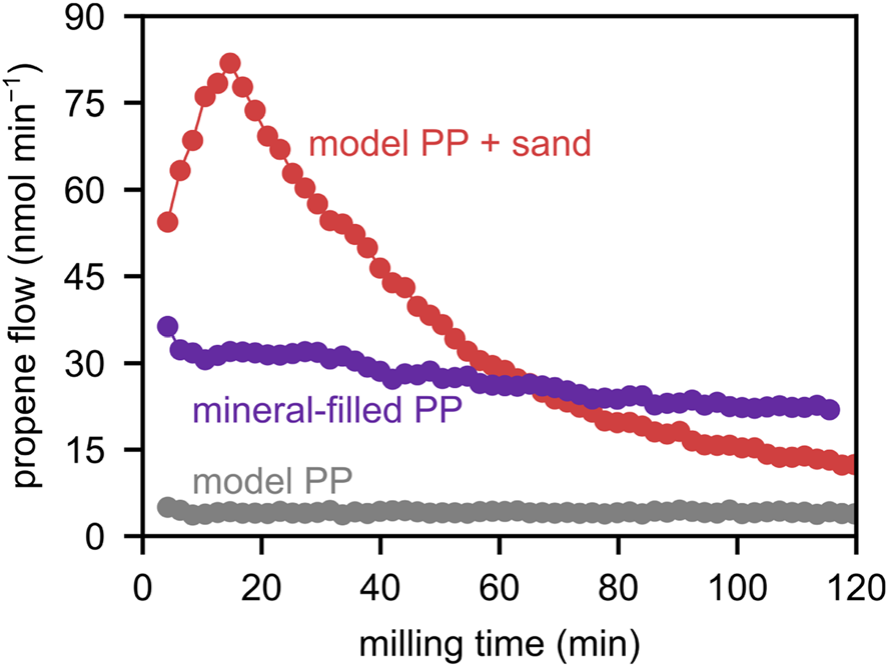
Propene flow during milling of 2 g of PP (denoted as model PP) with and without 1 g of sand and milling of 2 g of mineral-filled PP with 5 ZrO_2_ spheres at 30 Hz.

Inorganic materials, such as found in glass fiber-reinforced or mineral-filled plastics are typically unwanted in (chemical) recycling scenarios since they can, for example, scratch reactor equipment due to their hard nature.^30,31^ Under mechano-chemical conditions, however, we showed that inorganic materials such as sand can actually promote depolymerization. We performed ball milling experiments with ground garden furniture made from PP that contains such inorganic additives, typically 5–20% talcum, chalk or silicates. The depolymerization rate from this feedstock is much higher than that of laboratory-grade PP, illustrating the beneficial effect of these additives/impurities (Fig. 2 and S8), and offering a promising chemical recycling strategy for mineral-filled polyolefins.

To understand why the addition of sand increases the production of small hydrocarbons during milling of PP by a factor of 24.9 ± 1.2, we formulate several hypothetic mechanisms that could explain the observations:

(i) Milling with sand catalytically activates the grinding spheres.

(ii) Sand increases the temperature and causes thermo-chemical cracking of the polymer.

(iii) Existing paramagnetic impurities in sand promote radical depolymerization.

(iv) Radicals formed by fracture of sand initiate depolymerization or stabilize polymer mechano-radicals.

In the following, we discuss evidence regarding these hypotheses in more detail.

### Hypothesis (i): milling with sand catalytically activates the grinding spheres

The addition of hard sand to the grinding mixture could chemically alter the surface of the grinding spheres to increase their depolymerization activity. We have recently reported suitable strategies to incorporate mechano-catalytically active sites on the surface of ZrO_2_ grinding spheres.^10^ These active sites are defects that include partially reduced metal sites (Zr^3+^) and oxygen vacancies occupied by electrons (F-centers). These entities could offer favorable adsorption sites and stabilize polymer mechano-radical intermediates, which would ultimately translate to higher small hydrocarbon formation rates. In this context, milling with sand could introduce such defects on the grinding spheres by vigorous contacts with sand particles. In addition, these contacts could scratch the surface of grinding spheres to increase their roughness and surface area. This could promote the conversion of polymer material since reactions of molecules on solid surfaces generally benefit from maximizing the number of available active surface sites and thus an increased surface area. In addition, convex surface irregularities could focus mechanical impact forces in smaller domains, essentially increasing the density of mechanical energy input. Furthermore, higher surface roughness could increase friction in shear contacts and cause higher force transfer to the plastic material. However, although adding sand to the milling mixture indeed scratches the surface of the grinding spheres (Fig. S9), we believe that this mechanism is not the main driver of the observed boost in hydrocarbon productivity, based on the following two arguments.

**Fig. 3:**
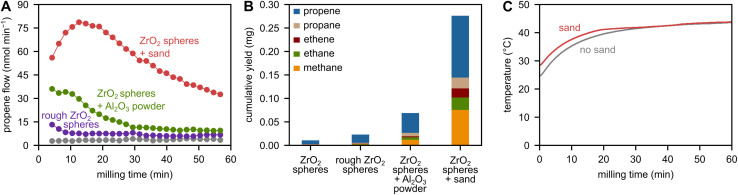
(A) Propene flow during milling of 2 g of PP with 5 ZrO_2_ spheres, either pristine (grey) or roughened by pre-milling with sand, at 30 Hz. And milling of 2 g of PP with 5 pristine ZrO_2_ spheres at 30 Hz with 1 g of sand or alumina. (B) C_1–3_ hydrocarbon yield obtained after 1 h of milling 2 g of PP at the same conditions as in panel A. (C) External container temperature during milling of 2 g of PP with 5 ZrO_2_ spheres at 30 Hz with and without 1 g of sand. Directly after milling with and without sand, the temperature inside the milling jar was measured to be 45 °C.

When comparing the Mohs hardnesses and Young's moduli (Table S1) of the materials, it becomes clear that ZrO_2_ grinding spheres are scratched much more easily by alumina (Mohs hardness 9) than quartz (Mohs hardness 7).^32–35^ SEM images of grinding spheres that have been milled with sand or alumina confirm the increased roughening induced by the higher hardness of alumina compared to sand (Fig. S9). Accordingly, milling with alumina powder should cause higher depolymerization rates than milling with sand due to the higher concentration of surface defects. This is, however, clearly not the case, with hydrocarbon productivity being much lower instead of higher when milling with alumina compared to sand (Fig. 3A and B), contrasting the hypothesis.

To investigate the role of surface imperfections created by contact of ZrO_2_ grinding spheres with sand particles, we pre-roughened grinding spheres by milling only with sand, and without PP. During subsequent milling of PP without sand, these grinding spheres are slightly more active than untreated spheres, but the activity is very far from milling directly with PP and sand (Fig. 3A and B). Therefore, we believe that the potential surface activation of grinding spheres has only a minor effect on hydrocarbon productivity.

### Hypothesis (ii): sand increases the temperature and causes thermo-chemical cracking of the polymer

Another hypothesis is that milling with sand causes high local temperatures which can drive the thermal degradation of PP to the observed products. This hypothesis is based on the idea that hard sand particles get impacted by mechanical forces, and that this input of mechanical energy can then heat up the surface of the material and/or grinding sphere. Mechanisms for this transfer of mechanical to thermal energy include plastic deformation, annealing of cracks, viscous heating, and friction.^36^

This could cause local high energy domains with a high temperature, a phenomenon which is often described in terms of hot spots in the mechano-chemical literature.^36,37^ Such hot regions could then cause thermo-chemical cracking of the plastic material. For an observable effect, hot spot temperatures must reach the onset of thermo-chemical cracking, which is *ca.* 300 °C for the PP used herein as determined by thermogravimetric analysis (Fig. S10).^10^ While hot spots can in principle reach temperatures of >700 °C and persist for up to tens of milliseconds,^36^ we believe that the reactivity of our system is not sufficiently captured by temperature effects based on the following four observations.

We measured the bulk temperature on the outside of the container during milling with and without sand (Fig. 3C). The depolymerization of plastic material is certainly not caused by bulk heating, since temperatures stay below 50 °C in both cases. Accordingly, we did not observe any C_1–3_ hydrocarbons when heating the container without shaking to 100, 160, or even 200 °C with a heating wire (Fig. S11). In addition, the temperature measured on the outside of the container during milling only increases initially and very mildly when milling with sand compared to without (Δ*T* < 5 °C), indicating not much of a thermal effect when adding sand. In addition, we measured the temperature inside the container directly after stopping the milling to be 45 °C with and without sand, which is only slightly higher than on the outside, indicating low bulk temperatures. Furthermore, we did not observe any melting of the polymer when milling with or without sand (Fig. S12), and it therefore seems unlikely that the melting temperature of PP (160 °C), let alone the cracking temperature (300 °C) was locally exceeded.

According to mechano-chemical theory, milling with harder compared to softer additives increases hot spot temperatures, since harder materials with higher Young's moduli dissipate heat in smaller volumes because they deform less, leading to higher energy densities and local temperatures.^36^ If the hot spot mechanism would be the driver of depolymerization, milling with harder additives should cause higher yields than milling with softer additives. However, milling with rather hard alumina (lower yields) and rather soft sand (higher yields, Fig. 3A, B and Table S1) clearly contrasts this hypothesis.

The formation of new surfaces during fracture in the ball mill consumes energy. This conversion of mechanical energy into surface energy competes with its conversion into thermal energy through the mechanisms described above.^38^ The balance between these conversion pathways depends, among other factors, on material properties such as brittleness. The extensive fracturing observed when milling brittle sand (Fig. S1) suggests that a significant portion of mechanical energy is consumed for fracture, thereby limiting the energy available for generation of high temperature domains.

The observed decline in hydrocarbon productivity at longer milling times seems inconsistent with a dominant thermo-chemical cracking mechanism. If localized high temperature domains were driving the reactivity, a correlation between those temperatures and the rates of hydrocarbon formation would be expected. However, heat generated by mechanical impacts on sand particles is very unlikely to decline as quickly over time as the hydrocarbon productivity does. In fact, the smaller sand particles obtained *via* fracture could concentrate impact energy in smaller volumes, potentially generating even higher localized temperatures. This mismatch illustrates the inability of a hypothetical thermo-chemical cracking mechanism to explain the observed reactivity.

While it is unlikely that milling with sand initiates thermal cracking, higher bulk or local temperatures when milling with sand could affect depolymerization equilibria by promoting the thermodynamically challenging depropagation step.^13^ However, while we acknowledge the potential presence of higher local temperatures when milling with sand compared to milling without, we believe that these are not the main driver of the observed boost in small hydrocarbon production.

### Hypothesis (iii): existing paramagnetic impurities in sand promote radical depolymerization

Certain species in the sand matrix could interact with mechano-radicals to assist their depolymerization. Sand is a natural material and contains impurities.^39^ Species that are especially relevant to mechano-chemical depolymerization are those that can bind mechano-chemically generated hydrocarbon radicals. This can stop radicals from recombining/terminating, or direct reactivity from these intermediates towards small hydrocarbon products. Examples for such species are partially reduced metal centers, such as Zr^3+^ and W^5+^, which can favorably interact with radicals *via* their unpaired electrons.^10^ It is therefore reasonable to expect similar beneficial interactions of mechano-radicals with partially reduced metal species in sand.

**Fig. 4:**
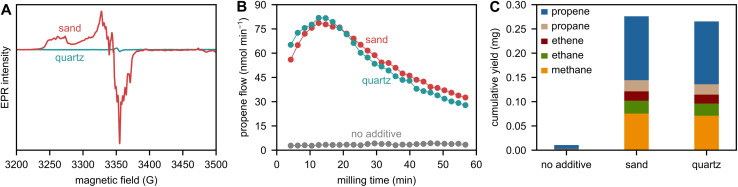
(A) Mass-normalized EPR spectra of sand and quartz, see Fig. S13 for magnified quartz spectrum. (B) Propene flow during milling of 2 g of PP with 5 ZrO_2_ spheres at 30 Hz with and without 1 g of sand or quartz. (C) C_1–3_ hydrocarbon yield obtained after 1 h of milling 2 g of PP with 5 ZrO_2_ spheres at 30 Hz with and without 1 g of sand or quartz.

We investigated the presence of such paramagnetic sites with unpaired electrons using EPR spectroscopy (Fig. 4A). Indeed, sand features a group of signals which are typical for aluminum species. These paramagnetic [AlO_4_]^0^ centers stem from the substitution of a silicon atom by an aluminum atom and charge compensation caused by an electron hole at a nearby oxygen atom, leading to an unpaired electron.^40,41^ In addition, an intense signal at *ca.* 3355 G indicates further paramagnetic species based on unpaired electrons at Si and/or O atoms (*vide infra*). However, we believe that the presence of inherent paramagnetic species in sand does not drive the observed boost in depolymerization. To judge the importance of these species for depolymerization, we performed reference experiments with laboratory-grade quartz, which does not feature significant levels of paramagnetic signals in EPR (Fig. 4A), but causes an identical boost in depolymerization activity compared to sand (Fig. 4B and C). Therefore, we conclude that inherent paramagnetic impurities in sand do not contribute to depolymerization.

### Hypothesis (iv): radicals formed by fracture of sand initiate depolymerization or stabilize polymer mechano-radicals

We believe that radicals generated from sand upon breakage direct the mechano-chemical conversion of PP towards small hydrocarbons. Fracture of covalent materials like sand has been reported to generate surface radicals by the cleavage of σ bonds within the material, which can lead to silicon-centered silyl (≡Si˙) and oxygen-centered siloxyl (≡Si–O˙) radical species.^15,17,18,25^

**Fig. 5:**
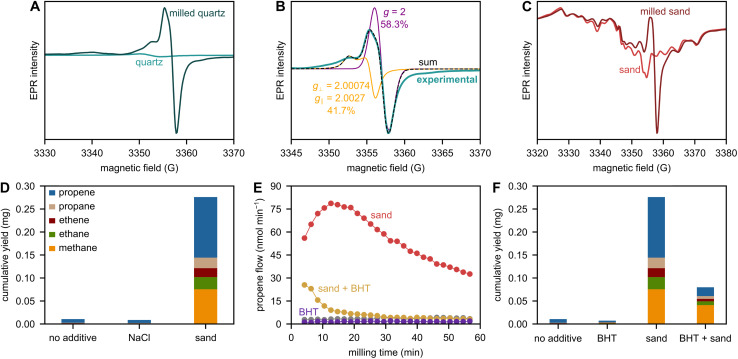
(A) Mass-normalized EPR spectra of quartz and milled quartz. (B) Simulation of experimental EPR spectrum of milled quartz with axially distorted component (41.7%) and isotropic component (58.3%). (C) Mass-normalized EPR spectra of sand and milled sand. (D) C_1–3_ hydrocarbon yield obtained after 1 h of milling 2 g of PP with 5 ZrO_2_ spheres at 30 Hz with and without 1 g of sand or NaCl. (E) Propene flow during milling of 2 g of PP with 5 ZrO_2_ spheres at 30 Hz without additive (grey) or with 1 g of sand or with 0.85 g of BHT or with 1 g of sand and 0.85 g of BHT. (F) C_1–3_ hydrocarbon yield obtained after 1 h of milling 2 g of PP with 5 ZrO_2_ spheres at 30 Hz without additive or with 1 g of sand or with 0.85 g of BHT or with 1 g of sand and 0.85 g of BHT.

To investigate the ability of sand and quartz to generate surface radicals upon fracture, we performed EPR spectroscopy. Milling of quartz (Fig. 5A) leads to a drastic increase in the signal amplitude and therefore concentration of paramagnetic species. We performed quantitative EPR spectroscopy *via* calibration of the instrument with paramagnetic CuSO_4_ and observed *ca.* 0.4 μmol g^−1^ of stable spins after milling quartz for 1 h at 30 Hz. We simulated the signal between 3345 and 3370 G as one axially distorted (*g*_⊥_ = 2.00074, *g*_∥_ = 2.0027) and one isotropic species (*g* = 2), see Fig. 5B. The axially distorted signal is likely caused by the presence of rather stable paramagnetic silyl radical species.^18,25,42^ The isotropic signal (*g* = 2) is likely part of a more complex pattern caused by oxygen-centered radical species, such as non-bridging oxygen hole centers (NBOHC, ≡Si–O˙) or peroxy species (≡Si–O–O˙). These oxygen-centered species could also be responsible for the component at 3340 G (*g* ≈ 2.01, Fig. 5A).^18,43^ However, such species would feature another signal at even lower magnetic fields (*g* = 2.05–2.08), which was not observed in our case, and it has been discussed that oxygen-centered species are more difficult to observe spectroscopically than silyl radicals.^25,43,44^ The presence of NBOHC species seems more likely than that of peroxy species due to the milling in nitrogen.^18,45^

The generation of paramagnetic radical species during milling of quartz was clearly shown by EPR. Paramagnetic species are also generated during milling of sand, which causes an increase in signal intensity between 3350 and 3360 G (Fig. 5C), likely related to similar species as detected for quartz. To show the importance of the presence of such surface radicals for depolymerization, we performed reference experiments with ionic NaCl, the fracture of which does not proceed *via* homolytic bond cleavage events, but only spatial separation of charged ions along a plane. Therefore, breaking of NaCl likely does not produce significant amounts of radicals. Indeed, milling PP with NaCl does not significantly increase small hydrocarbon formation (Fig. 5D). The same is true when adding hydrotalcite, a common additive used in plastics (Fig. S14).^46^ In addition to its ionic rather than covalent bonds which hamper radical generation and hydrocarbon formation, hydrotalcite is a layered material and very soft, and breaks by layers gliding past one another rather than through breaking covalent bonds. In contrast to NaCl and hydrotalcite, the activity of sand seems to be directly linked to its ability to form radicals upon fracture, and thus to the covalent nature of its crystal bonds.

We furthermore performed radical scavenging experiments using butylated hydroxytoluene (BHT). Adding BHT leads to the effective quenching of radical functionality in the milled mixture. BHT can react with surface radicals generated on sand upon fracture and with polymer radicals, which can abstract hydrogen atoms from the OH group of BHT. Hydrocarbon productivity is completely quenched after an initial induction period when milling with BHT and sand, indicating that radicals play an integral role in all product formation (Fig. 5E and F).

**Fig. 6:**
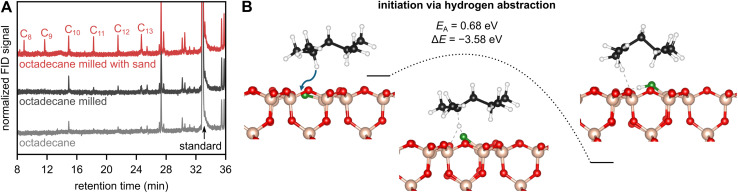
(A) Gas chromatograms recorded with a flame ionization detector (FID) before and after 1 h of milling 2 g of octadecane with 5 ZrO_2_ spheres at 30 Hz with and without 1 g of sand. (B) Reaction energy profile from DFT calculations for the abstraction of a hydrogen atom from a physisorbed DMP molecule by a surface of SiO_2_ containing O˙ as surface species.

To investigate the ability of surface radicals generated by fracture of sand to activate hydrocarbon substrates, we performed reference experiments with octadecane. Octadecane is used as a model compound, which is more defined and straight-forward to analyze compared to polydisperse PP. We milled octadecane with and without sand under an inert atmosphere and performed gas chromatography on the product mixture (Fig. 6A). Octadecane is a short hydrocarbon with no polymeric interactions, meaning that backbone bonds in octadecane cannot be cleaved mechano-chemically. It has been described that there is a limiting molecular weight below which the sum of van der Waals bonds are weaker than a backbone bond. Therefore, van der Waals bonds are broken rather than backbone bonds when exposing the material to mechanical forces. For polyethylene, a limiting degree of polymerization of 71–100 has been found,^4^ which is similar to that of other polymers,^12^ but much higher than the theoretical degree of polymerization of octadecane, which is 9. Accordingly, milling octadecane causes no backbone cleavage, and thus no shorter hydrocarbons are formed, as evident in the gas chromatograms (Fig. 6A).

**Scheme 1:**
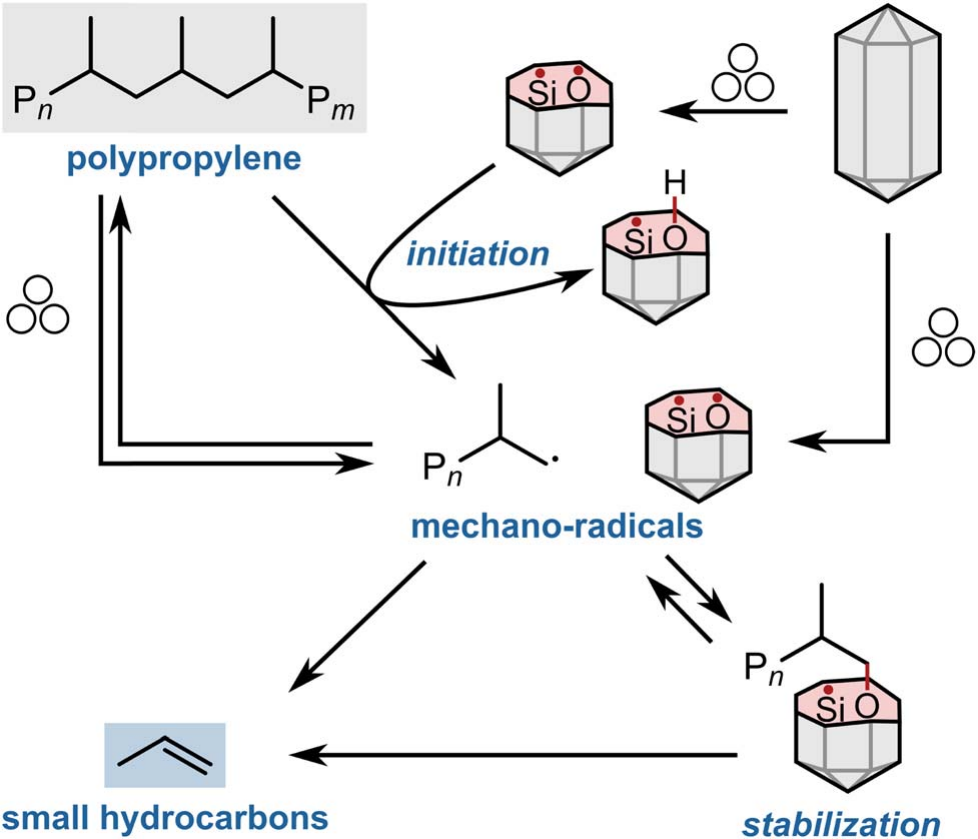
Proposed mechanism by which milling PP with sand increases small hydrocarbon yields. Fracture of sand crystals leads to silicon- and oxygen-based surface radicals, which can initiate reactivity by hydrogen abstraction from the polymer backbone, or stabilize polymer mechano-radical intermediates and direct their reactivity to small hydrocarbons.

In contrast, milling octadecane with sand does cause the formation of additional small hydrocarbons (Fig. 6A). The fracture of sand crystals seems to initiate reactivity. Mechanistically, we believe that surface radicals abstract hydrogen atoms from octadecane, which resembles the reported abstraction of hydrogen atoms from heteroatom-containing substrates by fractured quartz.^20,21^ Hydrogen abstraction from octadecane forms hydrocarbon radicals which can undergo follow-up reactions such as β scission or radical transfer reactions in combination with subsequent scission, leading to shorter hydrocarbons. It seems very plausible that identical mechanistic steps occur when milling with PP instead of octadecane, especially considering that abstraction of hydrogen atoms from PP can form tertiary alkyl radicals and therefore occurs more readily than from octadecane which forms secondary radicals. We therefore conclude that sand acts as a radical initiator (Scheme 1), and the increase in small hydrocarbon products seems to be at least partially rooted in the increase in hydrocarbon radical intermediates. In addition, we believe that stable sand and quartz becoming radical initiators under transient mechano-chemical conditions is interesting for a wider variety of applications. Using them as inorganic radical precursors could be beneficial in situations where explosive and difficult-to-handle azo compounds and peroxides are not practical, such as in the ball mill, where mechanical impacts could cause violent reactions.^47^

To further investigate the reactivity of surface radicals towards hydrocarbon substrates, we performed density functional theory (DFT) calculations.^48–55^ We formed silicon- and oxygen-based surface radicals on SiO_2_ by creating oxygen and silicon vacancies, respectively. According to our EPR spectra, we assumed that a representative surface contains both O˙ and Si˙ species. In addition, we investigated the effect of only O˙ present and only Si˙ present on the surface to understand their individual contributions to reactivity. Calculations were performed using 2,4-dimethyl pentane (DMP) as a model compound for PP. The physisorption of DMP on a surface containing O˙ and Si˙ causes only a slight stabilization of −0.62 eV (Table 1). In contrast, abstraction of hydrogen atoms from DMP leading to physisorbed secondary or tertiary hydrocarbon radicals is much more favorable (Δ*E* < −3.5 eV, Fig. 6B). With respect to selectivity, we found that the formation of tertiary radicals is more favorable than that of secondary radicals. The generation of primary radicals was also investigated, but those were thermodynamically not stable and were converted to secondary radicals during geometry optimization. The ability of fractured sand to abstract hydrogen atoms from DMP is in line with the experimental results obtained with octadecane and with its role as a depolymerization initiator for PP. Comparing hydrogen abstraction by surfaces containing only O˙ or only Si˙, respectively, shows that O˙ species seem to be the driving force of activity (Δ*E* < −3.5 eV), while hydrogen abstraction by Si˙ species is thermodynamically not favorable (Δ*E* > +2.4 eV).

**Table 1:** Reaction energies obtained by DFT for the reactivity of O˙ and Si˙ surface species on SiO_2_ towards DMP. Investigated pathways include physisorption of the unreacted molecule on the surface, hydrogen abstraction from DMP by the surface species to form a physisorbed hydrocarbon radical, and the subsequent stabilization of this hydrocarbon radical on the surface radical species. The denotation “not stable” is used for thermodynamically not stable products. Structures can be found in Tables S2–S4

Surface species	Δ*E* (eV)			
	Physisorption	Radical	Abstraction	Abstraction & stabilization
O˙ and Si˙	−0.62	Primary	Not stable	−5.23
		Secondary	−3.61	−4.42
		Tertiary	−3.97	−5.07
Only O˙	−0.43	Primary	Not stable	−4.93
		Secondary	−3.54	−4.60
		Tertiary	−3.99	−4.82
Only Si˙	−0.42	Primary	+3.63	Not stable
		Secondary	+3.19	Not stable
		Tertiary	+2.47	Not stable

In addition to their initiating role, we hypothesize that generated surface radicals could also stabilize polymer mechano-radicals that have been formed *via* either mechanical cleavage or hydrogen abstraction, and direct their reactivity towards small hydrocarbons. Radical intermediates could adsorb on silicon- and oxygen-based surface radicals (Scheme 1), which could have two effects: (i) sand-based adsorption sites could enhance the reactivity of polymer mechano-radicals to small hydrocarbons by favoring certain degradation pathways. For example, β scission or hydrogen transfer reactions could be favored compared to when free radicals react in the bulk medium. These degradation pathways could be radical in nature or not. A hypothetical example for the latter could be a series of β alkyl eliminations of the surface-bound species.^56^ (ii) Bonding of radicals could decrease their concentration and hamper termination reactions, *i.e.*, combination and disproportionation. Bonding could occur upon contact between hydrocarbon radical and sand surface radical, and radicals would be subject to an equilibrium between a free and a bound state. Re-activation from the bound state could either occur spontaneously or aided by mechanical impact. In the free state, β scission to monomers can occur. This mechanism is conceptually similar to controlled radical (de-)polymerization reactions where the reversible interaction of radicals with additives hinders termination reactions and enables more control over reactive intermediates.^57^ For example, after the generation of a pair of radicals *via* homolytic polymer backbone scission, one of them could adsorb on the SiO_2_ surface. Due to this stabilization, instantaneous recombination of the radical pair would be hampered, enabling the other radical to depolymerize more freely.

To investigate the interaction of hydrocarbon radicals with SiO_2_-based surface radicals, we extended our DFT calculations to include the adsorption of formed DMP radicals on surface radical species. Our results show the thermodynamic feasibility of adsorption of these radicals on O˙ species after formation (Δ*E* ≈ −4.5 eV for secondary, and Δ*E* ≈ −5.0 eV for tertiary radicals, Table 1), confirming the plausibility of the stabilization pathway in Scheme 1. Although we, therefore, expect the presence of C–O–Si species after milling PP together with sand/quartz, we could not observe these intermediates experimentally *via* Raman spectroscopy (Fig. S15). We believe that this is caused by the low concentration of such surface species compared to the predominant bulk polymer and quartz material.

To investigate the reactivity of adsorbed intermediates, we calculated the energy barrier (*E*_A_) for β scission from the adsorbed state (see Fig. S16 for reaction energy profile with local geometries of each state). The energy barrier was found to be *E*_A_ = 3.05 eV, which seems too high to justify the plausibility of β scission occurring directly from the adsorbed state. Therefore, we do not consider a direct catalytic effect of the SiO_2_-based surface radicals according to pathway (i) likely. In addition, during simulation, the polymer radical detaches from the SiO_2_ surface prior to scission, resembling a free β scission in the bulk rather than a catalytic surface reaction. Therefore, a hypothetical promotional effect of adsorption would not occur according to pathway (i) but rather according to pathway (ii). Fractured SiO_2_ would therefore not have a direct catalytic effect, but rather stabilize radical intermediates to hamper their termination, while depolymerization itself proceeds from free intermediates.

## Conclusions

Understanding the reactivity of polymer radicals generated upon ball milling is crucial to develop mechano-chemical conversion technologies. We show that increasing the number of radical intermediates is a powerful strategy to boost depolymerization yields and found that the homolytic fracture of added sand crystals forms surface radicals which react with polypropylene to form such intermediates. This results in an increase in polypropylene depolymerization yields by a factor of 25. In contrast, thermo-chemical cracking induced by localization of mechanical energy forming high temperature domains seems to have a negligible effect. Also surface activation of grinding spheres during milling and stabilization of mechano-radicals at partially reduced metal impurities in the sand matrix cannot explain the observed enhancement. In addition, milling with ionic materials does not affect the yield.

The transition of this technology to a larger scale would require adoption of a continuous rotary design. In addition, further understanding of the interplay of ball mill parameters, impact forces, and molecular reactivity is needed to increase depolymerization yields. For example, the effect of different types of impact forces, such as compression and shear, on the generation and reactivity of surface radicals must be studied.

While emerging mechano-chemical technologies promise energy efficient and selective depolymerization of plastics, they suffer from non-quantitative conversion and low yields due to the low reactivity of polymers at ambient temperatures. Our study shows that interfering with and promoting the radical pathway is a powerful concept to increase conversion and yield. In addition, we show that common inorganic impurities present in plastic waste will not necessarily lower depolymerization yields in mechano-chemical recycling processes, but can actually increase them. While the phenomenon of a limiting molar mass of the polymer below which ball milling cannot generate further radicals would limit mechano-chemical conversion, the ability of sand to induce radicals in polymers could be used to circumvent this conversion limitation.

## Author contributions

Conceptualization: IV; methodology: IV, AHH, RM, GL, CS; investigation: AHH, SP, RM, CLS; funding acquisition: IV; project administration: IV; supervision: IV; writing – original draft: AHH; writing – review & editing: AHH, SP, RM, CLS, CS, GL, IV.

## Conflicts of interest

There are no conflicts to declare.

## Supplementary Material

SC-016-D5SC03348A-s001

## Data Availability

Data for this article, including gas chromatography, electron paramagnetic resonance, X-ray diffraction, scanning electron microscopy and thermogravimetric analysis as well as analysis code are available *via* the Yoda repository at https://doi.org/10.24416/UU01-SJ5BKI. Experimental details, Fig. S1–S16, and Tables S1–S4. See DOI: https://doi.org/10.1039/d5sc03348a.
