# Supplementary material for: Homolytic fracture of inorganic crystalline materials enhances the mechano-chemical degradation of polypropylene
Source: Chem Sci. 2025 Aug 15;16(36):16511–21. doi: 10.1039/d5sc03348a (PMC12379351; doi:10.1039/d5sc03348a)
Supplement: SC-016-D5SC03348A-s001 [file SC-016-D5SC03348A-s001.pdf]

Supplementary Information (SI) for Chemical Science.
This journal is © The Royal Society of Chemistry 2025

Supporting Information

Homolytic fracture of inorganic crystalline materials enhances the mechano-chemical degradation of polypropylene

Adrian H. Hergesell[1], Stephan Popp[1], Raghavendra Meena[2], Viviana M. Ospina Guarin[1], Claire L. Seitzinger[1], Carsten Sievers[3], Guanna Li[2], Ina Vollmer[1]*

[1]Inorganic Chemistry and Catalysis Group, Institute for Sustainable and Circular Chemistry, Utrecht University, The Netherlands.

[2]Biobased Chemistry and Technology, Wageningen University, The Netherlands.

[3]School of Chemical & Biomolecular Engineering, Georgia Institute of Technology, Atlanta, Georgia, USA.

E-mail: i.vollmer@uu.nl

Experimental details

Ball milling experiments with polypropylene (denoted as (model) PP, Sigma-Aldrich, supplier values of M_n = 5,000 g mol^{-1} and M_w = 12,000 g mol^{-1}), industrial foil waste (PP/polyethylene foils with ca. 20% polyamide, and fractions of ethylene vinyl alcohol, polystyrene, and polyethylene terephthalate) or mineral-filled PP (PP with 5–20% talcum, chalk or silicates, obtained from ground garden furniture, density slightly above 1) were performed on a Retsch MM400 mixer mill at typically 30 Hz with five commercial ZrO_2 grinding spheres (10 mm diameter, Zhonglong Materials) or five commercial stainless steel grinding spheres (denoted as Fe, Retsch, X46Cr13 (1.4034), 12.5–14.5% Cr, 0.42–0.5% C) in a tungsten carbide container (Retsch, 25 ml). 2 g of PP were used together with 1 g of additive in a typical experiment. As additives, commercial sand (Fisher Scientific, general purpose grade, 40–100 mesh, pale brown very fine crystals < 0.5 mm), quartz (Merck, washed and calcined for analysis, 200–800 µm), Al_2O_3 (Alfa Aesar, 99.9% metals basis, α-phase, 20–50 µm), B_4C (3M, 212–300 µm), SiC (Alfa Aesar, 300–425 µm), hydrotalcite ($Mg_6Al_2(CO_3)(OH)_{16} \cdot 4\ H_2O$, synthetic, Sigma-Aldrich), and NaCl (Honeywell, 99%) were used. For radical quenching experiments, 0.85 g of BHT (2,6-di-*tert*-butyl-4-methylphenol, Sigma-Aldrich, 99%) were added to the container prior to milling. The preparation of pre-roughened grinding spheres (Al_2O_3 or sand) and pre-milled sand for ball milling experiments, scanning electron microscopy (SEM) and X-ray diffraction (XRD) was performed by milling 5.4 g of material with 5 ZrO_2 spheres for 1 h at 30 Hz.

Temperature measurements were performed by attaching a thermocouple to the outside of the container, securing it with adhesive tape. Directly after shaking stopped, one of the gas ports was opened and used to insert another thermocouple into the plastic material.

For reference experiments at higher temperature, a flexible glass yarn–insulated heating cable (Horst, 1.0 m, 100 W) was wrapped around the container. Subsequently, a thermocouple was attached to the container, and a woven fiberglass insulation tape was wrapped around and secured with heat-resistant adhesive tape.

To enable a continuous flow of 12.5 ml min^{-1} N_2 through the container and the analysis of hydrocarbon products, the container was equipped a gas inlet and outlet. To this end, holes were drilled into the commercial container via electrical discharge machining, and 1/8″ Swagelok connections were welded to it. Products were analyzed on an online Global Analyzer Solutions gas chromatograph, equipped with a thermal conductivity detector (TCD) and three flame ionization detectors (FID). The TCD was used for the detection of N_2 and H_2 and coupled to a 2 m × 0.32 mm Rtx-1, 3.0u and a 3 m × 0.32 mm Carboxen1010 column. For the detection of $C_{1–3}$ hydrocarbons, an FID coupled to a 3 m × 0.32 mm Rtx-1, 3u column

and a 15 m × 0.32 mm Al_2O_3/Na_2SO_4 column was used. For the detection of $C_{4–7}$ hydrocarbons, an FID coupled to a 2 m × 0.28 mm MXT-1, 1u column and a 14 m × 0.28 mm MXT-1, 1u column was used. For the detection of $C_{5–10}$ hydrocarbons, an FID coupled to a 2 m × 0.28 mm MXT-1, 0.5u column and a 15 m × 0.28 mm MXT-1, 0.5u column was used. The 12.5 ml min^{-1} flow of N_2 ($F_{\mathrm{N_2}}$) was used as an internal standard to account for potential changes in total volumetric flow ($F_{\mathrm{total},i} = \frac{F_{\mathrm{N2}}}{y_{\mathrm{N_2},i}}$) caused by the generation of gaseous products. **Eq. 1** was used to calculate the molar concentration of N_2 during each injection, using the average of the peak areas ($A_{\mathrm{N_2},i}$) of three stable injections before starting the reaction.

$$y_{\mathrm{N_2},i} = \frac{A_{\mathrm{N_2},i}}{\frac{\sum_{i=-2}^{i=0} A_{\mathrm{N_2},i}}{3}} \cdot \; y_{N_{2,0}} \tag{1}$$

The molar flow of a hydrocarbon C_xH_y with a carbon number x was determined according to **Eq. 2**.

$$F_{\mathrm{C}_x\mathrm{H}_y,i} = y_{\mathrm{C}_x\mathrm{H}_y,i} \cdot F_{\mathrm{total}} \cdot x \tag{2}$$

To calculate the concentration of a certain hydrocarbon, $y_{\mathrm{C}_x\mathrm{H}_y,i} = \frac{A_{\mathrm{C}_x\mathrm{H}_y,i}}{CF_{\mathrm{C}_x\mathrm{H}_y,i}}$ was calculated using its peak area $A_{\mathrm{C}_x\mathrm{H}_y,i}$ and calibration factor $CF_{\mathrm{C}_x\mathrm{H}_y,i}$. To determine the calibration factor, a calibration mixture with known contents of methane, ethane, propane, butane, heptane and hexane was used. The relationship $CF_{\mathrm{C}_x\mathrm{H}_y,i} = CF_{\mathrm{C}} \cdot x$ was utilized where CF_{C} is the calibration factor normalized by carbon number, since an FID response of a certain hydrocarbon is approximately proportional to its carbon number.

Cumulative yields of a certain hydrocarbon with a molecular weight of $M_{\mathrm{C}_x\mathrm{H}_y}$ were calculated according to **Eq. 3**, integrating its molar flow over time.

$$Y_{\mathrm{C}_x\mathrm{H}_y}[\mathrm{g}] = \frac{M_{\mathrm{C}_x\mathrm{H}_y}}{x} \cdot \int_0^{t_{\mathrm{final}}} F_{\mathrm{C}_x\mathrm{H}_y,i}\,\mathrm{d}t \tag{3}$$

Electron paramagnetic resonance (EPR) spectroscopy at 99 K was performed on a Bruker EMXplus instrument at an X-band microwave frequency of 9.4 GHz. A modulation frequency of 100 kHz and a modulation amplitude of 1 G were used. Reported spectra are normalized by mass. Milled sand and quartz were prepared by loading a modified 25 ml tungsten carbide container with 5.4 g of material and 5 ZrO_2 spheres, flushing for 20 min with 50 ml min^{-1} of N_2, closing the container with blind caps, and shaking for 15 min at 30 Hz. Samples were transferred to EPR tubes directly after milling and handled in air.

EPR spectra were simulated using the EPRsim Python package. One component was simulated as axially distorted (41.7%, $g_{\perp}$ = 2.00074, $g_{\parallel}$ = 2.0027, Gaussian linewidth of 0.8 G, Lorentzian linewidth of 1.22 G), while the other one was simulated as isotropic (58.3%, g = 2, Gaussian linewidth of 2.2 G, Lorentzian linewidth of 0.1 G).

To perform quantitative EPR experiments at 99 K, we calibrated the instrument using solutions of $CuSO_4 \cdot 5\ H_2O$ (Sigma-Aldrich) with different concentrations. We obtained a calibration line via linear fit to connect mass-based double integrals of the derivative spectra to spin concentrations in the sample. Finally, the spin concentration of milled quartz was calculated using this linear calibration. Measurements were performed less than 1 h after stopping the milling.

Thermogravimetric analysis (TGA) was performed on a Perkin Elmer TGA 8000 instrument under a 45 ml min^{-1} N_2 flow. Samples were heated from 50 °C to 600 °C with a heating rate of 10 °C min^{-1}.

Scanning electron microscopy (SEM) was performed on a Thermo Scientific Phenom ProX instrument using double-sided adhesive and conductive carbon tape on an aluminum holder. Images were recorded using an acceleration potential of 10 kV.

X-ray diffraction (XRD) was performed on a Bruker 2D PHASER instrument equipped with a LYNXEYE-2 detector using Cu K_{α} radiation (λ = 1.54056 Å) at 30 kV and 10 mA. Analysis was performed between 5 and 70° 2θ with a step size of 0.01°, a step time of 0.1 s.

Raman spectroscopy was performed on a Horiba Raman microscope using a 532 nm laser. Measurements were taken in a Raman shift region between 100 and 1800 cm^{-1}. Spectra were recorded using 10 scans, each one with a measurement time of 20 s, leading to 200 s of total acquisition time.

Offline gas chromatography (GC) after reactions with octadecane was performed on a Varian 430 gas chromatograph, using an Agilent CP9013 VF-5ms (30 m, 0.25 mm, 0.25 µm) column with a 10 m EZ-Guard column. The columns were placed in an oven with a program heating from 40 °C to 300 °C with a rate of 5 °C min^{-1} with an additional hold time of 10 min. An injection volume of 0.5 µl was used. To prepare samples, a modified 25 ml tungsten carbide container was loaded with 5 ZrO_2 grinding spheres, 2 g of octadecane (Sigma-Aldrich, 99%) and with or without 1 g of sand, flushed for 20 min with a 50 ml min^{-1} flow of N_2, and closed with blind caps. Subsequently, the container was shaken for 1 h at 30 Hz. Afterwards, the container was opened, the residue was solubilized in 1 ml of dichloromethane (Sigma-Aldrich, 99.9%), and 3 drops of butyl decanoate (Sigma-Aldrich, 98%) were added as internal standard prior to GC injection, but were not used for quantification.

Density functional theory (DFT) calculations[1,2] were performed with the Vienna ab initio Simulation Package (VASP.6.2.1)[3,4]. The generalized gradient approximation (GGA) with Perdew–Burke–Ernzerhof (PBE) exchange and correlation functional was used to account for the exchange–correlation energy.[5] The electron–ion interactions were described using the projected augmented wave (PAW) method and the plane-wave (PW) basis set.[3,4] The kinetic energy cut-off of the plane wave basis set was set to 520 eV. The convergence criterion for energy calculation and structure relaxation was set to a self-consistent field (SCF) threshold of 10^{-5} eV and a maximum force threshold of 0.05 eV/Å. The slab model for the most stable (001) α-quartz SiO_2 was obtained from Goumans *et al.* who used a thickness of 6 stoichiometric (18 atomic) layers of SiO_2.[6] However, for this work, we adopted the thickness to 4 stoichiometric (12 atomic) layers of SiO_2. The bottom two stoichiometric layers were fixed to mimic the bulk. A supercell of size 2 x 2 x 1 was used to obtain a sufficiently large surface. A Γ-centered k-mesh with size 1 × 1 × 1 was used for sampling the Brillouin zone of (001) α-quartz SiO_2. Gaussian-type smearing with a width of 0.05 eV was applied for the electronic energy density of states. Vacuum distances of 15 Å were added along the z direction to minimize interaction with the periodic images, and then dipole corrections were applied in the vacuum (z) direction. The van der Waals interactions were described by the DFT-D3BJ method developed by Grimme *et al.*.[7,8] The adsorption energies E_{ads} were calculated according to Eq. 4, where $E_{\text{slab+reactan}}$ is the total energy of the slab with a reactant adsorbed on it, E_{slab} is the total energy of the clean slab, and E_{reactant} is the total energy of the reactant. The adsorption energies were calculated as follows:

$$E_{\text{ads}} = E_{\text{slab+reactant}} - E_{\text{slab}} - E_{\text{reactant}} \qquad (4)$$

Here, $E_{\text{slab+reactant}}$ is the total energy of the slab with a reactant adsorbed on it, E_{slab} is the total energy of the clean slab, E_{reactant} is the total energy of the reactant.

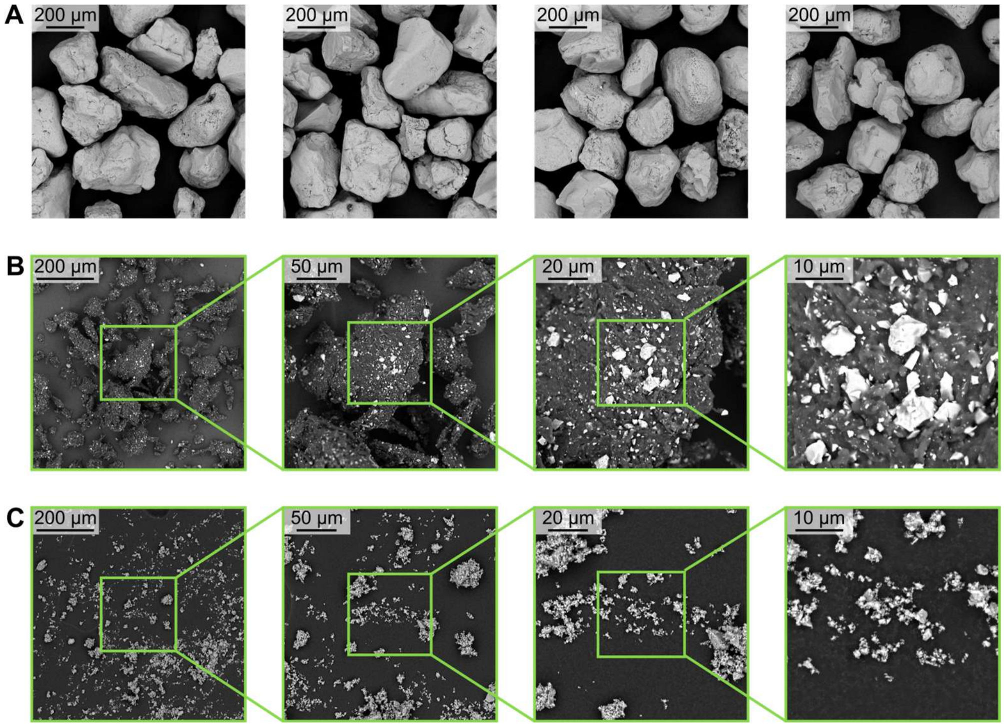

Fig. S1. (A) SEM images of untreated sand. **(B)** SEM images after 1 h of milling PP and sand with 5 ZrO_2 spheres at 30 Hz. The small sand particles appear much brighter than the larger PP particles due to the heavier elements contained. **(C)** SEM images of sand after 1 h of milling with 5 ZrO_2 spheres at 30 Hz.

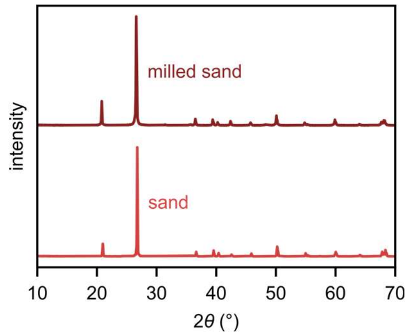

Fig. S2. X-ray diffractograms of sand before and after 1 h of milling with 5 ZrO_2 spheres at 30 Hz.

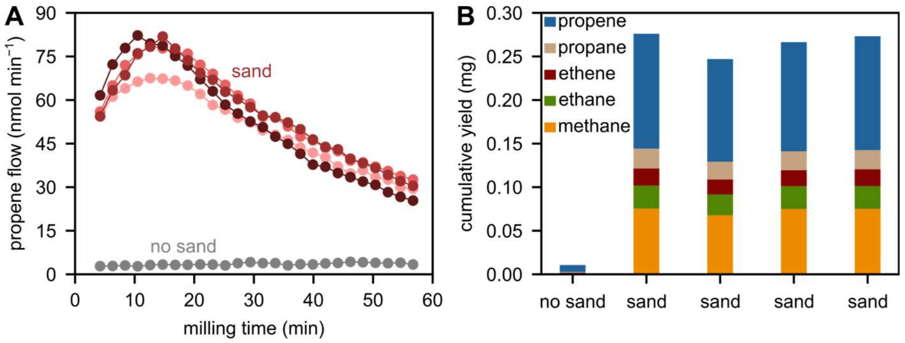

Fig. S3. (A) Propene flow during milling of 2 g of PP with 5 ZrO_2 spheres at 30 Hz with and without 1 g of sand (4 repetitions). **(B)** $C_{1–3}$ hydrocarbon yield obtained after 1 h of milling 2 g of PP with 5 ZrO_2 spheres at 30 Hz with and without 1 g of sand (4 repetitions).

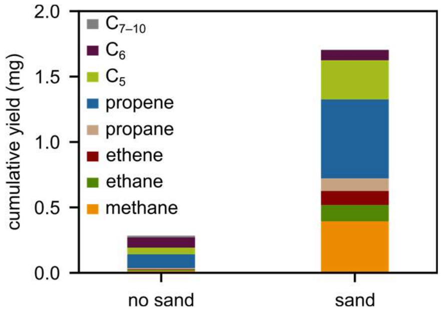

Fig. S4. $C_{1–10}$ hydrocarbon yield obtained after 0.5 h of milling 0.1 g of PP with 5 ZrO_2 spheres at 30 Hz with and without 1 g of sand. The increased yields compared to milling with a higher PP loading are likely related to more forceful contacts and the mitigation of the cushioning effect.[9]

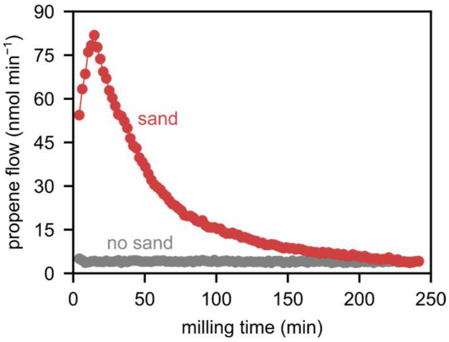

Fig. S5. Propene flow during milling of 2 g of PP with 5 ZrO_2 spheres at 30 Hz with and without 1 g of sand.

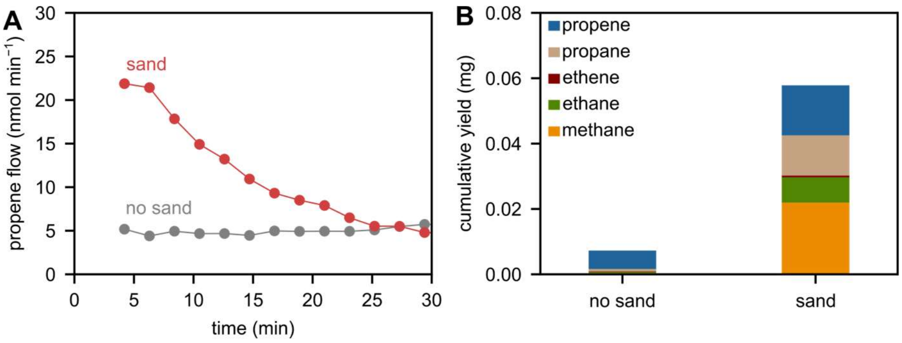

Fig. S6. (A) Propene flow during milling of 2 g of PP with 5 Fe spheres at 30 Hz with and without 1 g of sand. **(B)** $C_{1–3}$ hydrocarbon yield obtained after 0.5 h of milling 2 g of PP with 5 Fe spheres at 30 Hz with and without 1 g of sand.

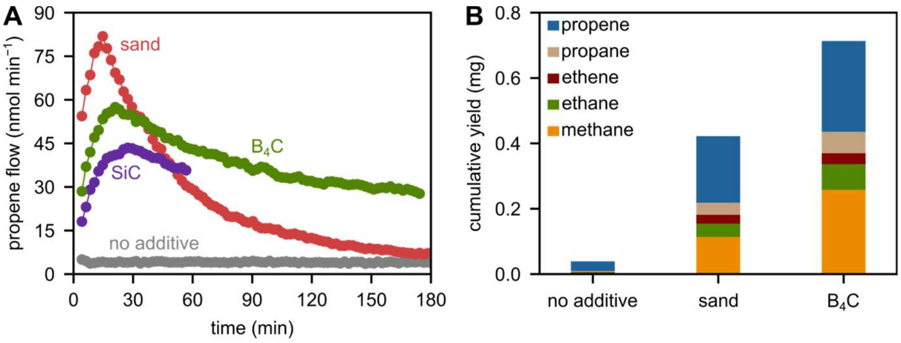

Fig. S7. (A) Propene flow during milling of 2 g of PP with 5 ZrO_2 spheres at 30 Hz with and without 1 g of sand, B_4C, or SiC. **(B)** $C_{1–3}$ hydrocarbon yield obtained after 3 h of milling 2 g of PP with 5 ZrO_2 spheres at 30 Hz with and without 1 g of sand, B_4C, or SiC.

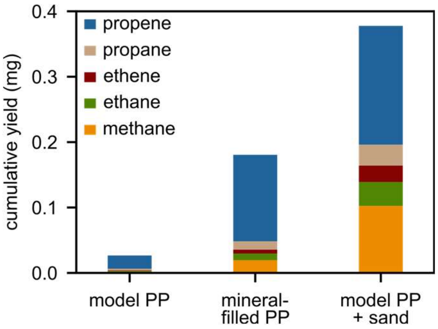

Fig. S8. $C_{1–3}$ hydrocarbon yield obtained after 2 h of milling 2 g of PP with and without 1 g of sand and milling of 2 g of mineral-filled PP with 5 ZrO_2 spheres at 30 Hz.

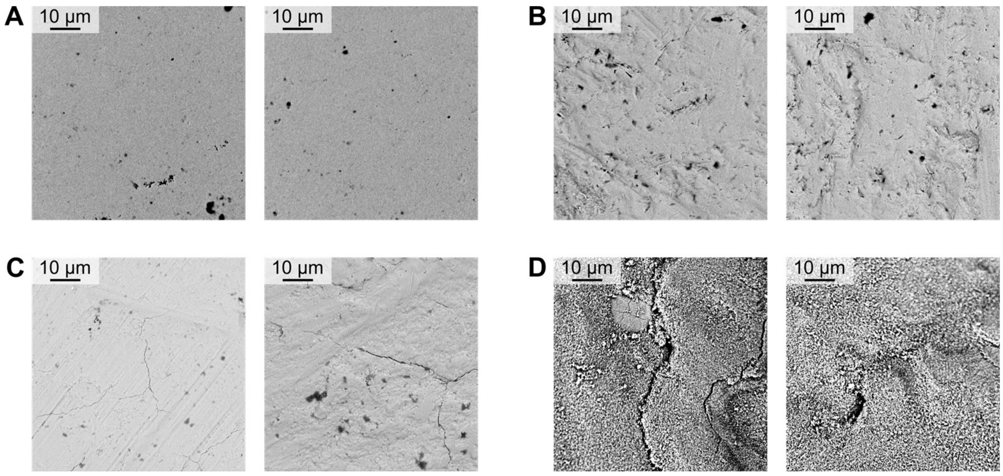

Fig. S9. SEM images of **(A)** a fresh ZrO_2 sphere, and a ZrO_2 sphere after milling with **(B)** PP and sand for 1 h at 30 Hz, **(C)** sand for 1 h at 30 Hz, and **(D)** alumina for 1 h at 30 Hz.

Table S1. Young's moduli *E* of PP, ZrO_2, Al_2O_3, and SiO_2.

	E (GPa)
PP	1.847[10]
ZrO_2	209[11]
Al_2O_3	390[11]
SiO_2	85.6[12]

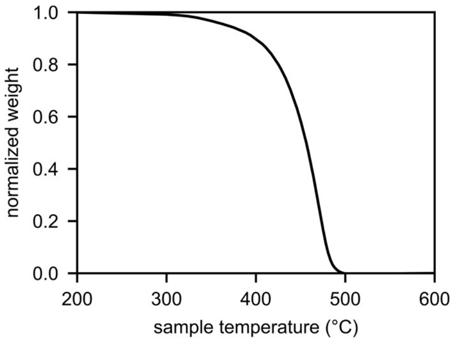

Fig. S10. TGA profile of PP. The onset of thermo-chemical cracking is ca. 300 °C.

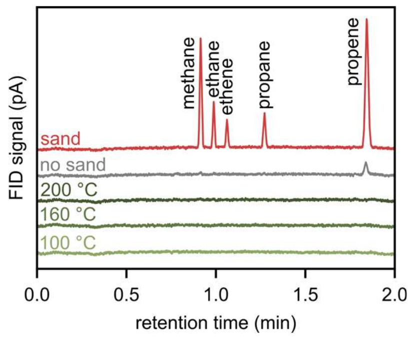

Fig. S11. Gas chromatograms recorded during milling of 2 g of PP with 5 ZrO_2 spheres at 30 Hz with and without 1 g of sand, and during heating of 2 g of PP and 1 g of sand without milling at 100, 160, and 200 °C.

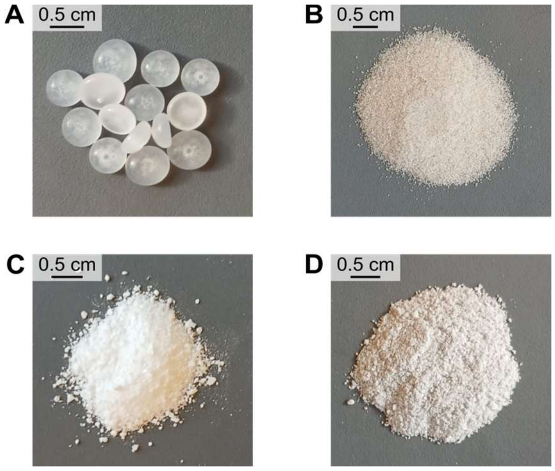

Fig. S12. Photographs of **(A)** pristine PP, **(B)** pristine sand, and **(C)** residue after 1 h of milling 2 g of PP with 5 ZrO_2 spheres at 30 Hz without and **(D)** with 1 g of sand.

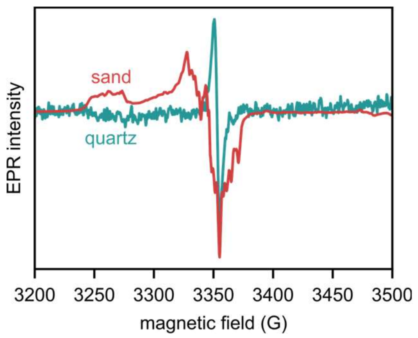

Fig. S13. Mass-normalized EPR spectra of sand and quartz. The quartz spectrum was magnified for comparison.

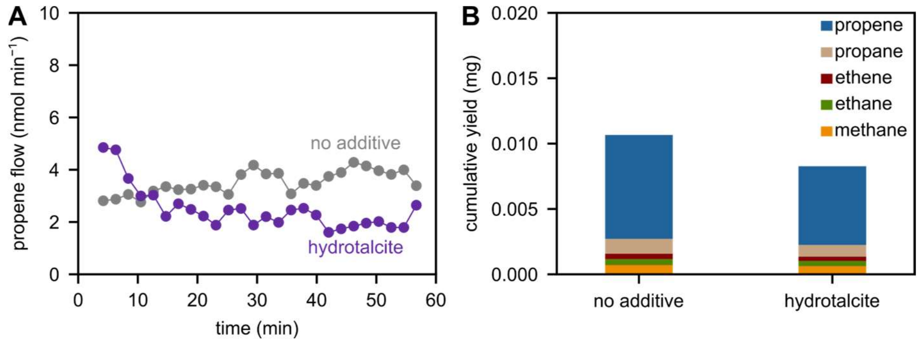

Fig. S14. (A) Propene flow during milling of 2 g of PP with 5 ZrO_2 spheres at 30 Hz with and without 1 g of hydrotalcite. **(B)** $C_{1–3}$ hydrocarbon yield obtained after 1 h of milling 2 g of PP with 5 ZrO_2 spheres at 30 Hz with and without 1 g of hydrotalcite.

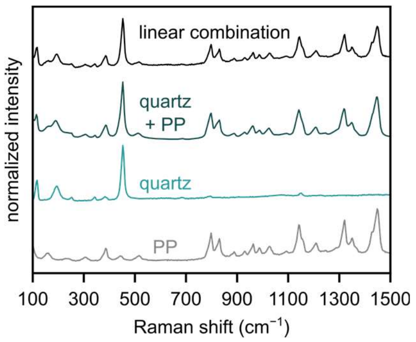

Fig. S15. Raman spectra of milled quartz, milled PP, a mixture of quartz and PP obtained after 1 h of milling 2 g of PP with 1 g of quartz with 5 ZrO_2 spheres at 30 Hz, and a virtually identical linear combination of the spectra of milled quartz and milled PP.

Table S2. Images of calculated geometries before and after reaction of DMP on SiO_2 containing O• and Si• as surface species.

clean surface	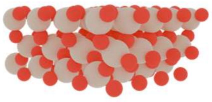	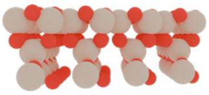	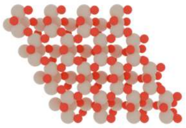
physisorption	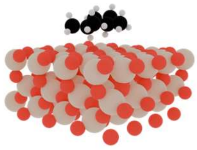	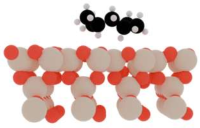	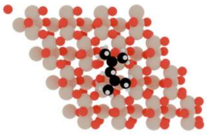
abstraction, primary	not stable	not stable	not stable
abstraction, secondary	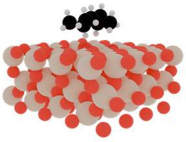	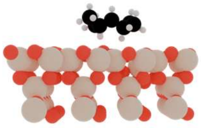	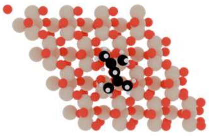
abstraction, tertiary	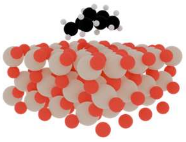	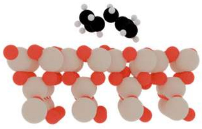	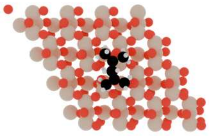
abstraction & stabilization, primary	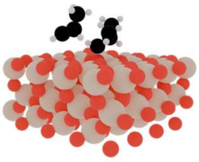	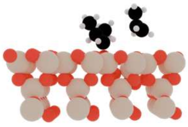	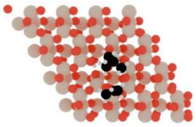
abstraction & stabilization, secondary	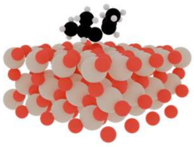	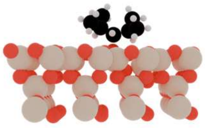	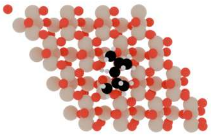
abstraction & stabilization, tertiary	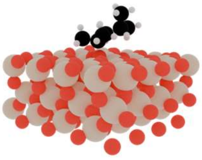	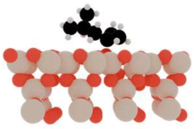	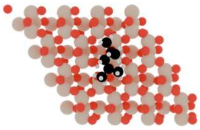

Table S3. Images of calculated geometries before and after reaction of DMP on SiO_2 containing O• as a surface species.

clean surface	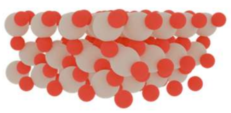	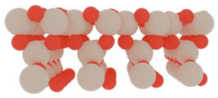	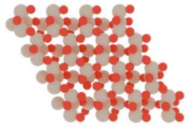
physisorption	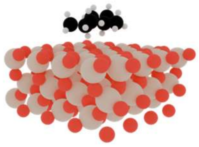	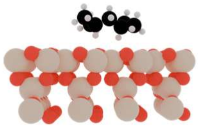	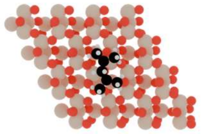
abstraction, primary	not stable	not stable	not stable
abstraction, secondary	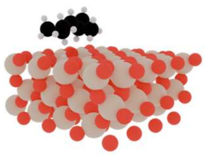	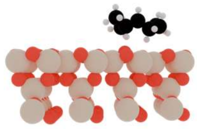	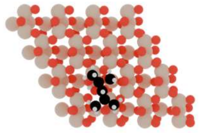
abstraction, tertiary	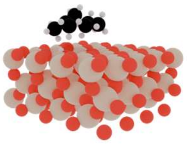	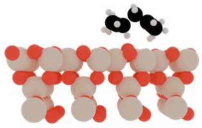	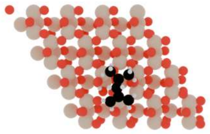
abstraction & stabilization, primary	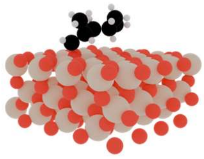	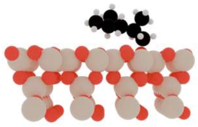	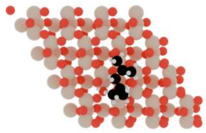
abstraction & stabilization, secondary	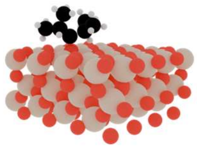	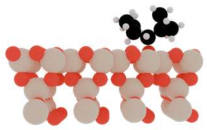	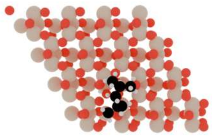
abstraction & stabilization, tertiary	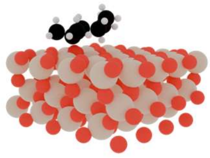	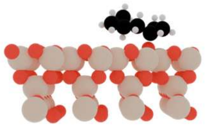	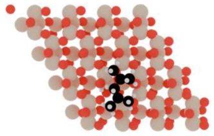

Table S4. Images of calculated geometries before and after reaction of DMP on SiO_2 containing Si• as a surface species.

clean surface	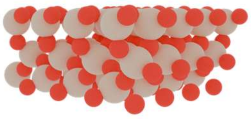	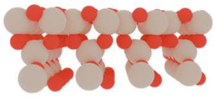	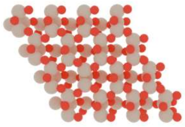
physisorption	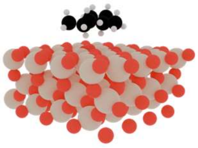	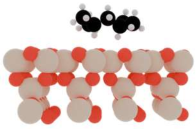	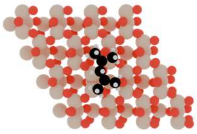
abstraction, primary	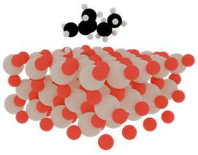	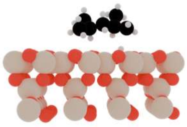	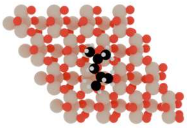
abstraction, secondary	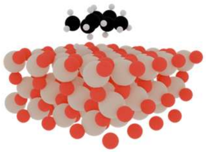	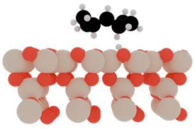	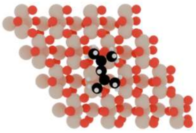
abstraction, tertiary	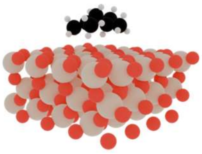	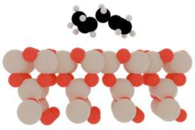	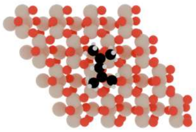
abstraction & stabilization, primary	not stable	not stable	not stable
abstraction & stabilization, secondary	not stable	not stable	not stable
abstraction & stabilization, tertiary	not stable	not stable	not stable

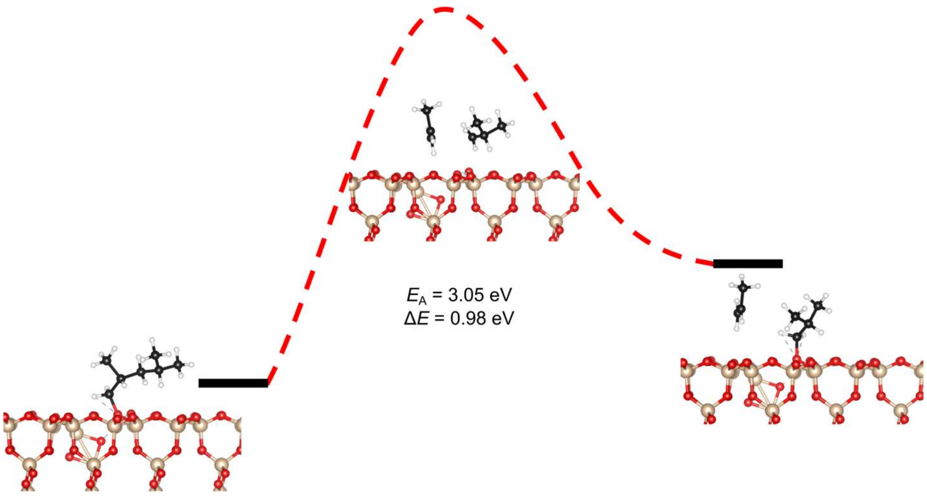

Fig. S16. Reaction energy profile from DFT calculations for the β elimination step from a primary DMP radical on SiO_2 containing O• and Si• as surface species.

References

1 P. Hohenberg and W. Kohn, Inhomogeneous Electron Gas, *Phys. Rev.*, 1964, **136**, B864–B871.

2 W. Kohn and L. J. Sham, Self-Consistent Equations Including Exchange and Correlation Effects, *Phys. Rev.*, 1965, **140**, A1133–A1138.

3 G. Kresse and J. Furthmüller, Efficient iterative schemes for ab initio total-energy calculations using a plane-wave basis set, *Phys. Rev. B*, 1996, **54**, 11169–11186.

4 G. Kresse and J. Furthmüller, Efficiency of ab-initio total energy calculations for metals and semiconductors using a plane-wave basis set, *Comput. Mater. Sci.*, 1996, **6**, 15–50.

5 J. P. Perdew, K. Burke and M. Ernzerhof, Generalized Gradient Approximation Made Simple, *Phys. Rev. Lett.*, 1996, **77**, 3865–3868.

6 T. P. M. Goumans, A. Wander, W. A. Brown and C. R. A. Catlow, Structure and stability of the (001) α-quartz surface, *Phys. Chem. Chem. Phys.*, 2007, **9**, 2146–2152.

7 S. Grimme, J. Antony, S. Ehrlich and H. Krieg, A consistent and accurate ab initio parametrization of density functional dispersion correction (DFT-D) for the 94 elements H-Pu, *J. Chem. Phys.*, 2010, **132**, 154104.

8 S. Grimme, S. Ehrlich and L. Goerigk, Effect of the damping function in dispersion corrected density functional theory, *J. Comput. Chem.*, 2011, **32**, 1456–1465.

9 A. H. Hergesell, C. L. Seitzinger, J. Burg, R. J. Baarslag and I. Vollmer, Influence of ball milling parameters on the mechano-chemical conversion of polyolefins, *RSC Mechanochemistry*, 2025, **2**, 263–272.

10 M. Kumar, K. K. Gaur and C. Shakher, Measurement of Material Constants (Young's Modulus and Poisson's Ratio) of Polypropylene Using Digital Speckle Pattern Interferometry (DSPI), *J. Japanese Soc. Exp. Mech.*, 2015, **15**, s87–s91.

11 M. Borba, M. D. de Araújo, E. de Lima, H. N. Yoshimura, P. F. Cesar, J. A. Griggs and Á. Della Bona, Flexural strength and failure modes of layered ceramic structures, *Dent. Mater.*, 2011, **27**, 1259–1266.

12 J. Robertson and M. I. Manning, Limits to adherence of oxide scales, *Mater. Sci. Technol.*, 1990, **6**, 81–92.
